# Role of EGFR in the Nervous System

**DOI:** 10.3390/cells9081887

**Published:** 2020-08-12

**Authors:** Roberta Romano, Cecilia Bucci

**Affiliations:** Department of Biological and Environmental Sciences and Technologies (DiSTeBA), University of Salento, 73100 Lecce, Italy; roberta.romano@unisalento.it

**Keywords:** EGF, EGFR, peripheral nervous system, central nervous system, neurons, brain, neurodegenerative disease

## Abstract

Epidermal growth factor receptor (EGFR) is the first discovered member of the receptor tyrosine kinase superfamily and plays a fundamental role during embryogenesis and in adult tissues, being involved in growth, differentiation, maintenance and repair of various tissues and organs. The role of EGFR in the regulation of tissue development and homeostasis has been thoroughly investigated and it has also been demonstrated that EGFR is a driver of tumorigenesis. In the nervous system, other growth factors, and thus other receptors, are important for growth, differentiation and repair of the tissue, namely neurotrophins and neurotrophins receptors. For this reason, for a long time, the role of EGFR in the nervous system has been underestimated and poorly investigated. However, EGFR is expressed both in the central and peripheral nervous systems and it has been demonstrated to have specific important neurotrophic functions, in particular in the central nervous system. This review discusses the role of EGFR in regulating differentiation and functions of neurons and neuroglia. Furthermore, its involvement in regeneration after injury and in the onset of neurodegenerative diseases is examined.

## 1. Introduction

**Figure 1:**
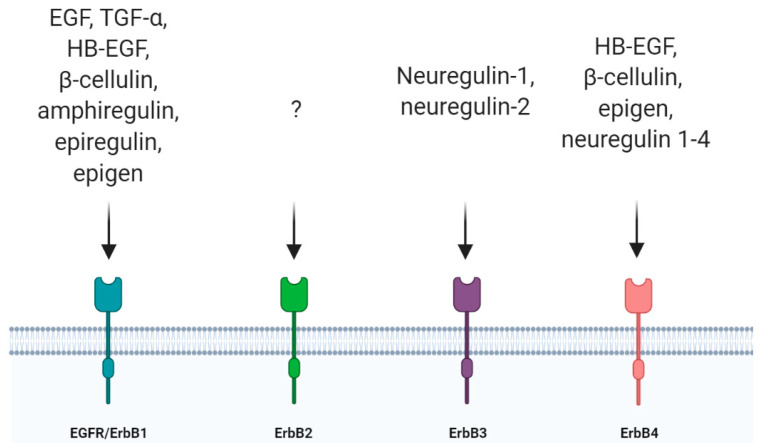
Representation of the four ErbB receptors and their ligands. EGF: epidermal growth factor; TGF-α: transforming growth factor-α; HB-EGF: heparin-binding EGF.

The epidermal growth factor receptor (EGFR, also known as ErbB1 or HER-1) belongs to the receptor tyrosine kinase (RTK) superfamily, which consists of other three members, ErbB2/Neu/HER-2, ErbB3/HER-3 and ErbB4/HER-4 [1,2]. EGFR was the first member of the family to be discovered [3] and, up to now, seven ligands are known to activate this receptor [4]. These ligands are structurally related proteins among which there are high-affinity ligands such as epidermal growth factor (EGF), transforming growth factor-α (TGF-α), heparin-binding EGF (HB-EGF) and b-cellulin (BTC), and low-affinity ligands such as amphiregulin (AR), epiregulin (EREG) and epigen (EPGN) [5]. Other ligands belonging to this family are represented by neuregulins (neuregulin 1–4) which bind only to ErbB3 and ErbB4 while ErbB2 is still an orphan receptor [6] (Figure 1). They all derive from integral membrane protein precursors that, after cleavage, give rise to their soluble forms containing a conserved three-loop compact structure, known as the EGF-like domain [7,8,9]. EGFR, like all RTKs, comprises an extracellular ligand binding domain, a single transmembrane domain and a cytoplasmic domain where there is a conserved protein tyrosine kinase core. EGFR homo- or hetero-dimerizes following ligand binding. Even if EGFR can homodimerize, the dimer formed by EGFR and ErbB2 constitutes the most active receptor, increasing the response to EGF [10,11]. As a result of the activation of the intrinsic kinase domain following ligand binding, specific tyrosine residues in the cytoplasmic tail are phosphorylated, becoming binding sites for adaptor proteins with Src-homology 2 domains (SH2) and activating downstream signaling pathways. Among these, the Ras-Raf-MEK-ERK1/2, STAT3 and STAT5 pathways and the PI3K-Akt-mTOR cascade are the main pathways activated by ligand binding to Erb receptors and they are fundamental in the regulation of cellular proliferation, differentiation and survival [1,2,12,13].

**Figure 2:**
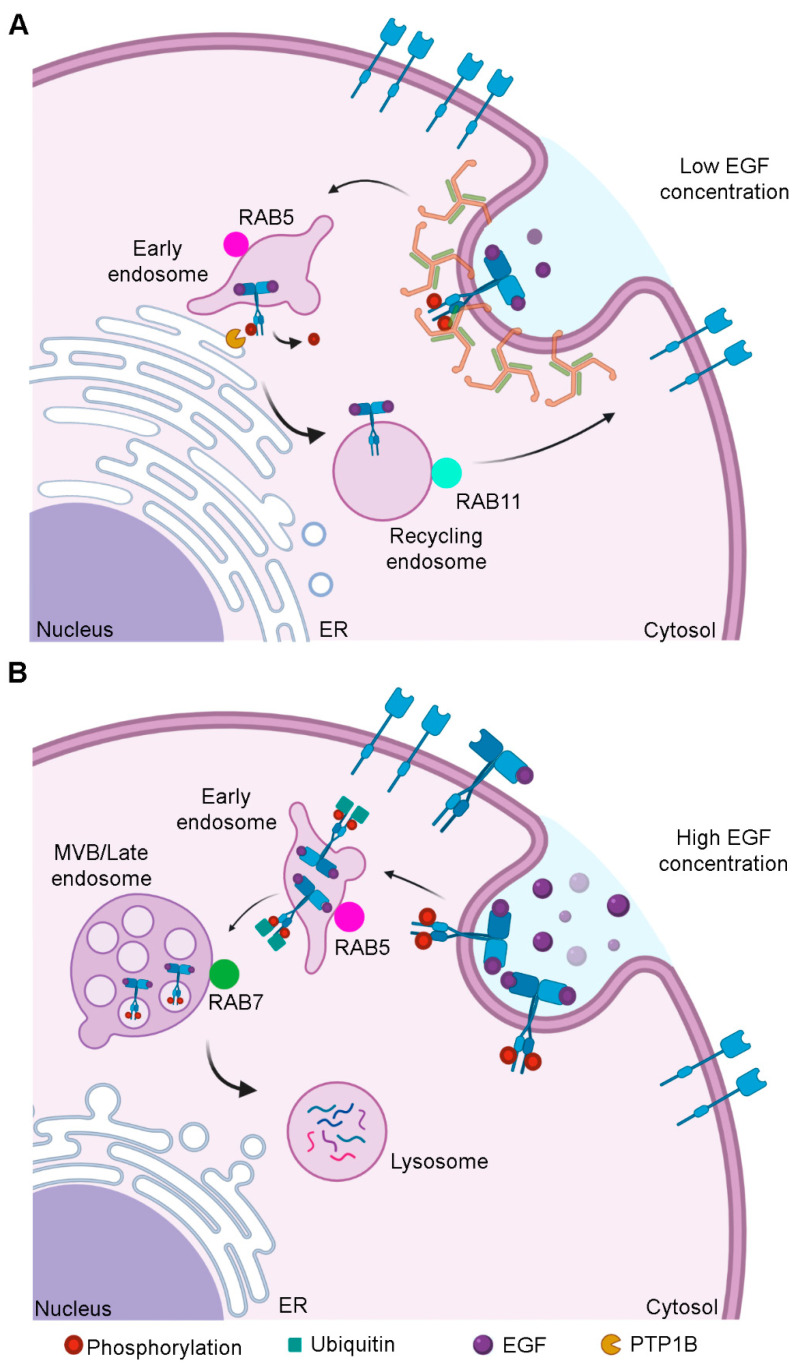
Intracellular fate of epidermal growth factor receptor (EGFR) after its activation. (**A**) When EGF is poorly concentrated, EGFR undergoes a low activation and it is subjected to clathrin-mediate endocytosis. The receptor reaches the early endosomes and tyrosine-protein phosphatase non-receptor 1 (PTP1B), which resides in the ER, dephosphorylates EGFR at the contact sites between ER and early endosomes. Then, EGFR is recycled back to the plasma membrane in RAB11-positive vesicles. (**B**) When EGF concentration is high, EGFR is more activated, and it is internalized through clathrin-independent endocytosis. After ubiquitination EGFR, reaches multivesicular bodies (MVBs) before being degraded into lysosomes.

The intracellular fate of EGFR depends on how it is activated (Figure 2). If the receptor undergoes a low activation, clathrin-mediated endocytosis occurs, so EGFR reaches RAB5-positive early endosomes that move toward the perinuclear region where EGFR is inactivated before being recycled back to plasma membrane by RAB11-positive recycling endosomes (Figure 2A). EGFR is inactivated as a result of the action of the tyrosine-protein phosphatase non-receptor 1 (PTP1B), which acts on the receptor at the ER-endosome contact-sites [14,15] (Figure 2A). On the contrary, if EGFR is highly activated, it is internalized through clathrin-independent endocytosis, it is not recycled but it reaches RAB7-positive late endosomes and then lysosomes in the perinuclear region where its degradation occurs [15] (Figure 2B). Degradation of EGFR is regulated by ubiquitination. Nonubiquitinated receptors are internalized through clathrin-mediate endocytosis and they are recycled back to plasma membrane rather than degraded [14]. This process is regulated by EGF concentration. In fact, when EGF is poorly available, activated EGFR is not marked for degradation [16]. Otherwise, the E3 ubiquitin ligase Cbl ubiquitinates EGFR, which is recognized by the ubiquitin-dependent adaptors of the endosomal sorting complexes required for transport (ESCRTs) and it reaches multivesicular bodies (MVBs), before being degraded into lysosomes [17,18].

MVBs originate from early endosomes by invagination of the limiting membranes which form intraluminal vesicles (ILVs). When endosomes accumulate ILVs in their lumen, they become MVBs [19]. After being incorporated into ILVs, molecules can be sorted towards three possible routes: they could be recycled, secreted through exosomes or degraded following the fusion of MVBs with lysosomes [20]. However, it has been demonstrated that MVBs can also behave as signaling organelles. In fact, in the case of the WNT pathway, WNT binds to both low-density lipoprotein receptor-related 6 (LRP6) and Frizzled receptors which polymerize before being internalized through caveolin containing vesicles [21]. The WNT receptor complex reaches the MVBs and this is an essential step to sustain WNT signaling [22]. It is still unknown if sequestration of cytosolic components in the MVBs to sustain signaling is required for other signaling pathways.

The EGFR pathway was initially described in *Drosophila melanogaster* and *Caenorhabditis elegans* and then better characterized through biochemical studies in mammalian cell culture [23,24,25,26].

**Figure 3:**
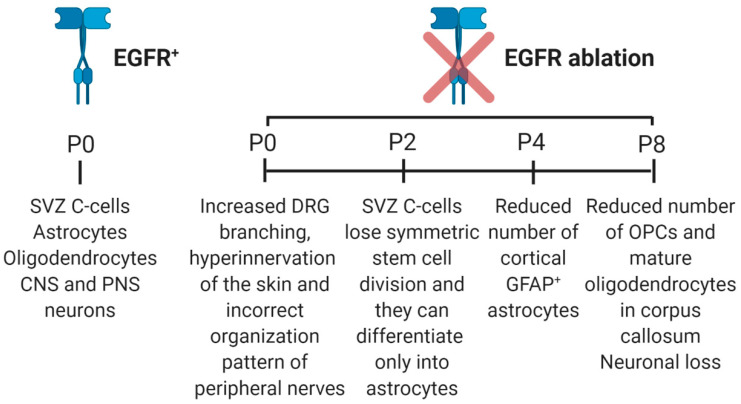
Diagram representing what happens to the nervous system cells after EGFR ablation. At P0, EGFR is widely expressed in the nervous system. EGFR deletion is detrimental for progenitors, astrocytes, oligodendrocytes and neurons. SVZ: subventricular zone; OPCs: oligodendrocyte precursor cells; PNS: peripheral nervous system; CNS: central nervous system; GFAP: glial fibrillary acid protein; DRG: dorsal root ganglia.

EGFR has important roles during development and in adult tissues of vertebrates [27]. In mammals, EGFR is crucial during embryogenesis, as it promotes embryo implantation and placenta development, and during organogenesis as it is involved in the development of several organs among which are the lungs, heart, bone, epithelia, kidney and liver [28]. For instance, in adults, EGFR has key roles in skin homeostasis as activation of its signaling pathway has an anti-apoptotic effect on keratinocytes affected by ultraviolet B radiation [29]. Furthermore, EGFR is fundamental for cellular proliferation and migration and for angiogenesis in skin wound healing [30]. Also, EGFR roles in kidney physiology have been well investigated and it was demonstrated that EGFR activation in epithelial cells of the proximal tube stimulates sodium reabsorption while activation of the receptor in the distal nephron reduces sodium reabsorption [31]. Therefore, EGFR is involved in growth, differentiation, maintenance and repair of various tissues and organs also including the nervous system. Indeed, EGFR can be found in the central nervous system (CNS) since astrocytes, oligodendrocytes, progenitor cells of the subventricular zone (SVZ) and some neuronal populations in the developing brain express it, while its expression has decreased in the adult brain [32,33,34,35,36]. EGFR is also expressed in the peripheral nervous system (PNS) and in particular in cutaneous nerves, sensory corpuscles, DRG (dorsal root ganglia) primary sensory neurons, satellite glial cells and Schwann cells [37,38,39,40]. Furthermore, EGFR-null mice grow with neural defects highlighting the key role of this receptor in the nervous system [41] (Figure 3).

In light of the recent findings about the importance of EGFR in the nervous system, the aim of this review is to summarize the current knowledge about its physiological role in the CNS and the PNS, its functions in regeneration after injury and its involvement in the onset and progression of nervous system diseases.

## 2. EGFR and the Nervous System

In the brain and in the nervous systems specific growth factors affect proliferation, differentiation and migration of neurons. Indeed, a fundamental role in these processes in neurons is accomplished by neurotrophins that use neurotrophin receptors to initiate cell signaling and regulate neuronal processes [42,43,44,45]. Thus, for a long time the presence and the role of EGFR in the nervous systems and in neurons has been underestimated.

The presence of EGFR in the brain has already been observed in 1988 in rats [46]. Indeed, EGFR immunoreactivity was found in astroglia 16 days after birth, reached a maximum 3 days later to then became weak or absent in adult rats. Interestingly, EGFR was present in the cerebral cortex of adult and aged rats and, in particular, EGFR-positive neurons were more present in the motor area of the frontoparietal cortex while they were less numerous in the somatosensory area [46]. Thus, it was hypothesized that EGF was not involved in astroglia proliferation during development but possibly participating in neuronal survival or signaling between neurons and glia cells [46]. Furthermore, the same authors demonstrated that while brain astroglia in adult rats express low levels of EGFR, after injury astrocytes showed high EGFR expression suggesting that EGFR may be involved in the transition to reactive astrocytes [46].

In 1994 it was described that EGFR was present in the skin, not just in the perineurium and Schwann cells, as expected, but also in axons of nerve bundles, in axon and lamellar cells of Meissner corpuscles and within the axon, inner core, outer core and capsule of Pacinian corpuscles [38]. These data proved that human cutaneous nerves and sensory corpuscles normally express EGFR [38].

Thus, in the past 25 years the role of EGFR in the nervous system has been investigated and its importance for neurons has been established although many aspects have not yet been clarified. In particular, while it is clear that EGFR in the CNS has multiple key roles complementing the role of neurotrophin receptors, its functions in the peripheral nervous system have been poorly investigated up to now.

## 3. EGFR Functions in the CNS

### 3.1. Role of EGFR in Neural Stem Cell Pool Maintenance

Neurogenesis occurs in restricted areas throughout adult life denying the dogma by which neurons cannot be generated in the adult brain. This process can take place because of the existence of adult neural stem cells (NSCs), multipotent cells which generate neurons and glial cells. NSCs are localized in specific niches of the lateral ventricle and the dentate gyrus of the hippocampus, the subventricular zone (SVZ) and the subgranular zone (SGZ) [47]. It has been demonstrated that EGFR is important for proliferation of NSCs derived from the mice embryo germinal zone since it is required for the formation of a high proliferative daughter-cell population holding the same stem cell-like characteristics of the mother cell [48,49].

More recently, a third proliferative region, the subcallosal zone (SCZ), was discovered. This region is localized between the corpus callosum and dorsal hippocampus and contains cells that in the presence of EGF can grow in vitro as clonal aggregates of cells called neurospheres and they are able to produce oligodendrocytes, neurons and astrocytes [50]. Not only SCZ but also SVZ cells are responsive to EGF. In fact, SVZ cells express EGFR and form multipotent and selfrenewing neurospheres if grown in vitro in the presence of EGF. Most of these cells responsive to EGF do not derive from quiescent stem cells in vivo but from rapidly dividing transit-amplifying C cells [51]. Otherwise, NSCs isolated from the fourth ventricle or spinal cord of adult mice produce neurospheres in vitro only if they are treated with a combination of fibroblast growth factor 2 (FGF2) + heparin or EGF + FGF2 [52]. In vivo, the infusion of FGF2 + heparin + EGF into the fourth ventricle increases cellular proliferation in the fourth ventricle and in the spinal cord. EGF alone is not able to stimulate proliferation while FGF2 + heparin have an effect on cells in the fourth ventricle but not in the spinal cord. Thus, only when EGF is added to FGF2 and heparin, an increase in proliferation is visible in both the fourth ventricle and the spinal cord. Considering that cells of the fourth ventricle do not express EGFR, cells expressing FGF2-receptor start to proliferate responding to FGF2 stimulation and then they upregulate EGFR to begin EGF-induced proliferation [53,54].

**Figure 4:**
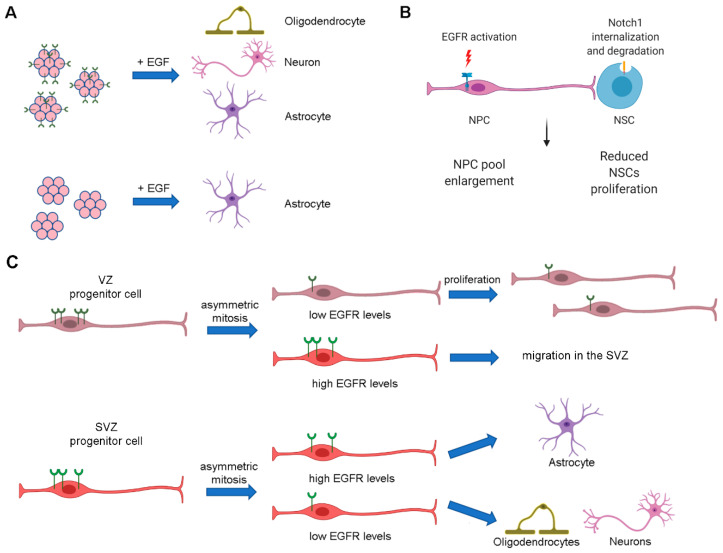
EGFR regulates neural stem cells and progenitors. (**A**) Neural stem cells can grow in vitro as neurospheres. These clonal aggregates can differentiate into oligodendrocytes, neurons or astrocytes if they express EGFR while, following EGFR ablation, they can differentiate only into astrocytes. (**B**) Following cell-cell interaction, EGFR activation in neural progenitor cells (NPCs) stimulates Notch1 ubiquitination and degradation in adult neural stem cells (NSCs). This mechanism is responsible for NPC pool enlargement at the expense of NSC proliferation. (**C**) In the VZ, NPCs expressing high EGFR levels can undergo asymmetric mitosis producing a daughter cell expressing low EGFR levels which continues to proliferate and a daughter cell expressing high EGFR levels which migrates in the SVZ. Here, it can divide asymmetrically producing a daughter cell with high EGFR levels that differentiates into astrocyte and a daughter cell with low EGFR levels that enters in a diverse differentiation path.

In order to understand if the neurodegeneration that occurs in mice lacking EGFR is due to a defect in the regulation of neural stem cells, the effect of EGFR depletion on stem cells derived from SVZ was evaluated [55]. Cells isolated from the SVZ of P2 EGFR knock-out mice, show complete ablation of EGFR, loose, in vitro, symmetric stem cell division that characterized selfrenewal but also display alterations in the capability of differentiate, since wild-type neurospheres are able to produce astrocytes and neurons, while neurospheres not expressing EGFR are able only to differentiate into astrocytes (Figure 4A). These results demonstrate the importance of EGFR in NSCs selfrenewal and that loss of EGFR signaling in cells derived from SVZ leads them to differentiate preferentially into glia [55]. Consistently, in adult SVZ, EGFR is specifically expressed by undifferentiated precursors (the transit-amplifying cells or C-cells), which are characterized by the absence of neuronal or glial markers [51,56]. On the contrary, in the adult dentate gyrus of hippocampus, where proliferation is less intensive compared to SVZ, the C-cells population is less present according to the reduced EGFR immunoreactivity and to the low or absent proliferation in response to EGF stimulation [57].

In order to guarantee supply of different cell type cells to the CNS, the balance between NSCs and neural progenitor cells (NPCs) has to be maintained [58,59,60]. EGFR and Notch1 pathways are important for the maintenance of NSCs and NPCs in the brain, revealing the existence of functional interactions between these two cellular populations. Increased EGFR signaling in neural precursor cells in vivo is associated with the enlargement of NPCs pool at the expense of NSCs, reducing their number, proliferation and selfrenewal capability. Notch1 is responsible for the maintenance of NSCs identity and selfrenewal [61,62]. EGFR activation on NPCs stimulates Notch1 ubiquitination on NSCs following cell-cell interaction and, consequently, its degradation therefore reducing neural stem cell proliferation [61,62] (Figure 4B). Another important factor regulating NPCs selfrenewal is Sox2 [63,64]. Indeed, a feedback loop involving Sox2 and EGFR exists to enhance NPCs selfrenewal [65]. In fact, activation of EGFR signaling stimulates Sox2 expression and Sox2 can bind to the EGFR promoter, thus upregulating EGFR expression [65]. EGFR is downregulated by Sox2 knockdown while it is upregulated by Sox2 overexpression and this fosters NPCs’ selfrenewal [65].

Neural-cell diversity is also guaranteed by asymmetric mitosis. NSCs and progenitors can be subjected to asymmetric cell divisions producing two different cell daughters, a copy of itself and a committed progenitor that enters in the path of differentiation [66,67]. Asymmetric mitosis also concerns the cell surface receptor since EGFR can distribute in an asymmetric way during mitosis in mouse embryonic forebrain ventricular and subventricular zones, in vitro and in vivo. The daughter cell expressing high levels of EGFR is committed to differentiate into an astrocyte while the daughter cell with low EGFR levels does not express astrocyte markers, suggesting a different fate (Figure 4C) [68]. In fact, in the SVZ, progenitor cells more inclined to differentiate into glia than into neurons are characterized by increased EGFR expression [69]. Furthermore, cell proliferation, migration and differentiation into astrocytes are stimulated by EGFR activation in late progenitors cells which move from the ventricular zone to the SVZ in EGFR-dependent manner [70].

Altogether these results indicate that EGF and EGFR signaling play important roles in neural stem cells and progenitors being able to regulate both selfrenewal and differentiation.

### 3.2. Role of EGFR in Astrocyte Differentiation and Maturation

Astrocytes represent about 40% of all cells in the CNS and they are important to sustain neuronal function [71]. Astrocytes are derived from radial glial cells that, after a “gliogenic switch”, generate immature astrocytes which, in turn, migrate away from ventricular zone heading for cortical layers where they divide few times before exiting the cell cycle [72].

It has been demonstrated that the choice between proliferation and differentiation depends on ligand concentration and on the age of progenitor cells [70]. EGFR is important to regulate differentiation of precursors into astrocytes since SVZ neural progenitor cells expressing low EGFR levels differentiate into neurons or oligodendrocytes while high EGFR levels determine their differentiation into astrocytes [70]. Indeed, EGFR can be asymmetric distributed during late stage of mitosis of progenitor cells producing a cell daughter with high EGFR levels that is committed to becoming an astrocyte [73]. Recently it was demonstrated, through yeast two-hybrid screening, that EGFR interacts with necdin, a protein highly expressed by postmitotic neurons and that interacts with nuclear proteins to suppress mitosis of proliferative cells and promote neuronal survival [74]. In primary cortical progenitor cells it was shown that necdin interacts with EGFR in its active state and is able to inhibit astrocytes differentiation induced by EGFR activation in vitro, bringing to light a new EGFR-dependent mechanism underlying gliogenesis [75].

Immature astrocytes migrate from the ventricular zone and mature in the cortical layers, but this process is still poorly understood. Recently, it was demonstrated that the EGFR effect on astrocytes’ maturation is based on a positive feedback loop. In fact, maturing astrocytes produce HB-EGF, one of the EGFR ligands, and express its receptor so it has an autocrine function, inhibiting their maturation [76,77]. Withdrawal of HB-EGF from immature astrocytes decreases HB-EGF expression, similarly to what happens following EGFR inhibition, while increasing HB-EGF or enhancing EGFR signaling leads to further increases in HB-EGF levels and therefore promotes the immature astrocyte state [78]. This positive feedback loop could amplify a small decrease in HB-EGF levels by further decreasing HB-EGF production, which leads to astrocyte maturation. Consistently, a gradual decrease of HB-EGF expression as astrocytes mature occurs [78]. Interestingly, astrocytes cultured in vitro for more than 3 weeks become refractory to EGF and do not proliferate anymore. However, when these astrocytes are pretreated with interleukin-6, they return to be responsive to EGF and resume the cell cycle suggesting a synergic action between cytokines and EGF [79].

**Figure 5:**
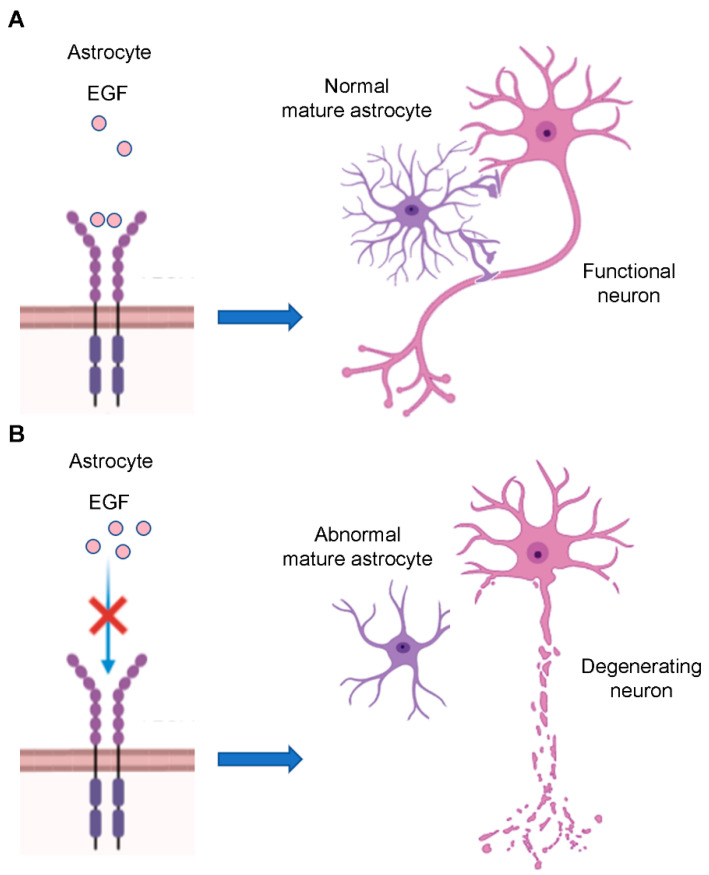
EGFR regulate astrocyte morphology. (**A**) Mature astrocytes show cribriform structures which surround axons sustaining neuronal functions. EGFR signaling stimulates the acquisition of these processes. (**B**) EGFR blockade during CNS development inhibits the formation of astrocytes’ processes leading to neuronal degeneration.

EGFR acts also on astrocytes’ morphology. To sustain neuronal function, astrocytes acquire a particular morphology represented by cribriform structures that encircle axons. Immature astrocytes show few processes, which are instead numerous in mature astrocytes. EGFR is also important for the acquisition of these structures since blockade of its signaling during CNS development results in disorganization of astrocytes that lose their processes surrounding neurons, leading to degeneration of many axons in the optic nerve (Figure 5A,B) [80,81].

The supporting role towards neurons played by EGFR-expressing astrocytes is also suggested by accumulation of abnormal astrocytes in EGFR-knockout mice forebrains where massive apoptosis of neurons occurs [82,83,84]. Interestingly, EGFR^−/−^ midbrain astrocytes are unaffected while EGFR^−/−^ cortical astrocytes activate apoptosis in an Akt- and caspase-dependent manner, losing their capability to sustain neurons, as it was demonstrated in coculture experiments [85]. Interestingly, although midbrain and cortical astrocytes express equivalent levels of EGFR and its ligands, expansion and survival of midbrain astrocytes are not dependent on EGFR signaling, thus they could be regulated by other signaling pathways, suggesting the existence of functionally different population of astrocytes in the mouse midbrain and cortex [85]. This result could explain why in EGFR^-/-^ mice, neurodegeneration occurs in the cortex and olfactory bulbs but not in the other areas of the brain [86]. Indeed, in vivo studies on several mouse strains, demonstrate that, in EGFR-knockout mice, degeneration of the frontal cortex, olfactory bulbs and thalamus observed at P4 results in loss of extensive parts of the brain at P8 and this phenotype could be explained by a reduced number of GFAP (glial fibrillary acid protein)-positive astrocytes which are also less proliferative in vitro [86].

So, EGFR signaling could be necessary for neuronal survival in a cell-autonomous manner, since neurons express the receptor, but it could also stimulate astrocytes to secrete neurotrophic factors required for the survival of neurons. Consequently, the reduced number of astrocytes in EGFR^−/−^ mice may not support neurons efficiently, contributing to the neurodegeneration observed. The reduced number of astrocytes could be also explained with the effect of the lack of EGFR on neural progenitors since it was demonstrated that increased EGFR signaling correlates with a greater ability of progenitor cells to differentiate into astrocytes [70]. But the fact that FGF2 alone is sufficient to induce stem cells differentiation into astrocytes could explain why there is a reduction but not a complete block of astrocyte differentiation in the absence of EGFR [87]. Altogether these data indicate that EGF and EGFR signaling affect different steps of astrocyte differentiation and maturation, being fundamental for the biology of these cells.

### 3.3. Role of EGFR in Oligodendrocyte Maturation

In the CNS oligodendrocytes are responsible for the generation of myelin sheaths that surround axons allowing axonal survival and saltatory conduction of nerve impulses [88].

**Figure 6:**
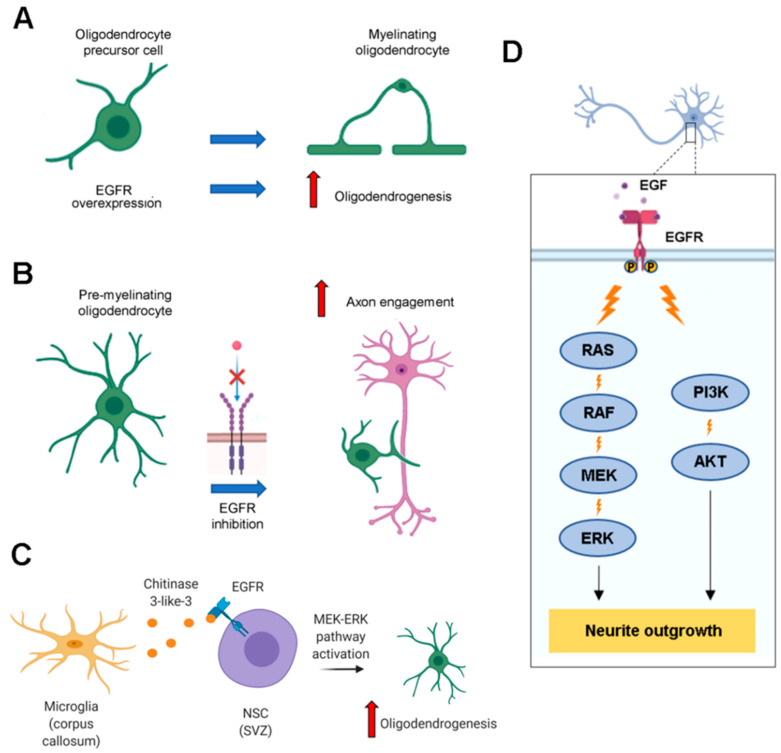
EGFR regulates oligodendrogenesis and neurite outgrowth. (**A**) EGFR overexpression stimulates oligodendrocyte precursor cells to differentiate into myelinating oligodendrocytes, highlighting its important role in oligodendrogenesis. (**B**) Axon engagement, which represents the final step of oligodendrocytes maturation, is stimulate by EGFR inhibition. (**C**) Microglia in the corpus callosum produce chitinase-3-like-3 which activates EGFR expressed by NSCs in the near SVZ. This leads to MEK/ERK pathway activation that promotes oligodendrogenesis. (**D**) EGFR activates AKT and ERK signaling pathways which are important for neurite outgrowth.

EGFR is involved in oligodendrocytes’ maturation during development. In fact, EGF treatment stimulates differentiation of bipotential oligodendrocyte precursor cells (OPCs) into myelinating oligodendrocytes [89]. Furthermore, EGFR overexpression increases the number of differentiated myelinating oligodendrocytes, the expression of myelin basic protein (MBP) in the corpus callosum and the number of myelinated axons two weeks after mouse birth, highlighting the important role of EGFR in oligodendrogenesis and myelin production (Figure 6A) [90].

However, it is not clear at what stage of oligodendrogenesis EGF starts to carry out its functions. Starting from mice tripotential glial-restricted precursor cells (GRPs) which give rise to OPCs, isolated from E13.5 spinal cord and cultured, it has been demonstrated that cells belonging to the oligodendrocytes lineage are responsive to EGF at all developmental stages [91]. EGF is able to stimulate GRPs and OPCs proliferation and selfrenewal but this effect seems to be related to the increased response to platelet derived growth factor-AA (PDGF-AA) promoted by EGF and not to a direct inhibition of their differentiation [91]. In fact, EGF alone stimulates GRPs differentiation in OPCs and finally in mature oligodendrocytes [91].

EGF-induced activation of EGFR causes increased Akt phosphorylation which is involved in myelination during early embryonic stages [92]. SHP-2, a Src-homology 2 domain (SH2)-containing tyrosine phosphatase, has key roles in generation, proliferation and myelination of oligodendrocytes in vivo and it can activate Akt in OPCs in an EGFR-dependent manner [93,94,95]. Oligodendrocytes’ maturation induced by SHP-2 is impeded following treatment with specific Akt inhibitors, highlighting the role of the EGFR-signaling pathway in oligodendrocytes’ maturation in the embryonic brain [95]. Another Akt partner is represented by Grb2 associated binder1 (Gab1), an intracellular signaling mediator for many growth factor and cytokine receptors, such as EGFR, that amplifies signals leading to a stronger activation of downstream pathways like phosphatidylinositol 3-kinase (PI3K) pathway [96,97]. It was found that EGFR-dependent Gab1/Akt activation contributes to expansion of Olig2^+^ progenitor cells in the developing spinal cord suggesting that EGFR modulates Olig-2 expression through Gab1 and Akt and therefore regulates oligodendrogenesis [98].

While oligodendrogenesis requires the upregulation of EGFR signaling, the final step of oligodendrocytes maturation, that is axon engagement, is fostered by EGFR inhibition (Figure 6B). In fact, it was demonstrated that EGFR inhibition stimulates MBP expression in premyelinating oligodendrocytes and cotreatment with Clobetasol and Gefitinib, which inhibit EGFR, favors MBP expression and localization of phosphatidylinositol 4,5-bisphosphate (PIP2) in endosome-like structures leading to membrane expansion and, consequently, to axon engagement [99].

In the adult brain, NSCs present in the SVZ are able to differentiate into OPCs that migrate in the site of injury and mature into myelinating oligodendrocytes. Under physiological conditions, the production of OPCs which migrate into the corpus callosum to mature in myelinating oligodendrocytes is low [100]. However, if a demyelinating insult occurs [101], NSCs start to give rise to OPCs that migrate in the white matter and it is known that EGFR signaling is a stimulus to oligodendrogenesis [90,102].

Activation of NSCs is not cell-autonomous but depends on the SVZ microenvironment [103,104,105]. It was demonstrated that microglia, localized in the corpus callosum near the SVZ and the lateral ventricle, produces chitinase 3-like-3 which functions in the CNS are still unknown. This factor binds to EGFR expressed by NSCs and activates the MEK/ERK signaling pathway leading to oligodendrogenesis in vitro [106] (Figure 6C). Generally, neurons represent the cell type in which SVZ precursors differentiate more but EGF infusion in the brain causes a strong proliferation of precursors that migrate in the parenchyma around the SVZ into the striatum, septum, corpus callosum and fimbria-fornix and then differentiate into oligodendrocytes, while neuronal differentiation does not occur [107].

EGFR expression is reduced as cells advance along the differentiation pathway [108] and constitutive EGFR activation in oligodendrocyte progenitors is associated with diffuse hyperplasia in postnatal white matter [109]. Thus, EGFR seems to be downregulated in oligodendrocyte lineage in the adult but, after a demyelinating damage, factors produced in the SVZ by microglia stimulate NSCs to differentiate into OPCs and EGFR expression and activation in oligodendrocyte lineage stimulates oligodendrogenesis to repair the damage, recapitulating what happens during development.

Altogether, these data demonstrate that EGF and EGFR signaling have a key role in oligodendrocyte strongly influencing different steps of maturation.

### 3.4. Role of EGFR in Neurite Outgrowth

Axon elongation and dendrite branching are essential for nervous system function. They are controlled by signals produced by neurons or other cell types. Interestingly, axonogenesis can be influenced by EGF-stimulated astrocytes and EGFR activation creates an environment promoting axonal growth [80]. Indeed, neurite outgrowth from retinal explants is limited following EGF treatment and in the absence of astrocytes in vitro, while cocultures of astrocytes and neonatal retinal explants demonstrate that neurite outgrowth is greater and faster following EGF administration [80].

EGFR is also expressed by neurons and its activation regulates neurite outgrowth. Phosphorylation, induced by EGF or by suppressor of cytokine signaling 2 (SOCS2) overexpression, stimulates neurite outgrowth in PC12 cells and cortical neurons [110,111]. Another mechanism leading to neurite outgrowth involves EGFR, tissue kallikrein (TK) and flotillin-2. In fact, TK stimulation facilitates activation of EGFR which forms a complex with flotillin-2 leading to ERK 1/2 activation and, as a consequence, to an increase of neurites number and their mean length in SH-SY5Y cells and primary neurons [112]. Recently, additional data corroborate the role of EGFR in neurite outgrowth. In particular, it was found that EGFR, stimulated by EGF, is involved in dendrite branching in the early stage of development and this is mediated by Huntingtin-interacting protein 1-related protein (HIP1R), which is necessary to EGF-induced early endocytosis of EGFR [113]. Akt and ERK are the main signaling pathways activated by EGFR and they are involved in neurite outgrowth (Figure 6D). Regarding Akt, two recent works highlighted its role in promoting neurite outgrowth [114,115]. The first demonstrated that Akt pathway activation by insulin is able to attenuate neurite outgrowth impairments induced by arsenic [114]; the second showed that Streptozotocin can be beneficial for neuronal cells inducing neurite outgrowth through activation of PI3K-Akt signaling pathway [115]. This is a *N*-nitrosourea natural compound discovered in a strain of *Streptomyces achromogenes* and originally identified as an antibiotic [116] On the other hand, it was found that the G3 domain of versican, one of the major extracellular matrix (ECM) proteins in the brain, stimulates ERK phosphorylation, enhancing neurite growth of hippocampal neurons [117].

Hydroxymethylglutaryl–coenzyme A (HMG-CoA) reductase is a key enzyme for cholesterol synthesis [118] and it was demonstrated that HMG-CoA reductase inhibitor mevastatin triggers neurite outgrowth in Neuro2a cells and this is accompanied by increased phosphorylation of EGFR, ERK 1/2 and Akt. Inhibition of this kinase cascade prevents neurite outgrowth after mevastatin treatment indicating that EGFR activation is necessary to mevastatin-induced neurite outgrowth [119].

Therefore, in CNS, EGFR activation stimulates downstream pathways important for neurite outgrowth during development and in mature neurons.

## 4. EGFR Functions in the PNS

Although the role of the ErbB family has been better investigated in the CNS rather than in the PNS, it has been demonstrated that these receptors are important for PNS development. In fact, early development of Schwann cells, which are glia cells responsible for production of myelin sheath wrapping sensory and motoneurons axons [120], is compromised by mutations in ErbB2 mouse gene. Mutant mice exhibit a peripheral neuropathy characterized by a thinning of the myelin sheaths [121]. Another study shows that mutations in ErbB2 receptor in mice embryos lead to defasciculated motor and sensory axons and to loss of Schwann cells in the peripheral nerves [122]. ErbB3 is also relevant for PNS development since mice lacking this receptor show loss of Schwann cells. Nevertheless, at the early stage of development they display normal motor and sensory neurons which, however, later on then degenerate [123].

Therefore, there are many reports regarding the role of other members of the ErbB receptor family in PNS development while little is known about the role of EGFR, even though it was demonstrated its expression in Schwann cells and sensory neurons [124].

Williams and co-workers identified, in the DRG at E12.5 in mouse, a population of progenitors of PNS expressing EGFR using in vitro clonal sphere-formation assay. During normal development, these cells derive from the neural crest and then differentiate into Schwann cells, smooth muscles/fibroblasts-like cells or, less often, neurons [125].

The first evidence of the importance of EGF in the development of PNS came in 2009 when it was demonstrated that this soluble factor was able to stimulate the differentiation of embryonic neural crest cells into neurons and melanocytes, highlighting the involvement of the EGFR signaling pathway in PNS [126]. In vivo experiments showed that EGFR-null mice display hyperinnervation of the skin by postnatal day 0 and nerves do not show the correct organized pattern. Furthermore, skin-targeted EGFR mutants do not display alteration in innervation suggesting that hyperinnervation is due to a defect in DRGs [127]. In fact, the same study demonstrated in vitro that hyperinnervation observed in EGFR null mice is due to increased axon branching establishing a role of EGFR in limiting neurite outgrowth and branching during PNS development [127].

EGFR also has a role in PNS physiology. The PNS comprises nerve fibers important for pain perception [128]. Recently, it was shown that EGFR is involved in pain processing. In particular, EGFR activation induced by epiregulin but not by other ligands stimulates nociception in mice. Moreover, EGFR knockdown in *Drosophila* sensory neurons impaired thermal nociception which is rescued by reintroduction of EGFR, demonstrating that EGFR regulates peripheral nociception in vivo [129].

Recently, we discovered a new form of Charcot-Marie-Tooth type 2B (CMT2B) disease, a peripheral neuropathy caused by a novel mutation (K126R) in the *RAB7* gene [130]. This form of CMT2B is characterized by a motor phenotype in contrast with the previously discovered forms caused by RAB7 mutations (L129F, N161T/I, V162M) that caused a predominantly sensory phenotype. Interestingly, in patient cells we found impaired EGFR degradation in contrast to the previously described forms that display normal or increased EGFR degradation [130,131,132,133]. Considering the expression pattern previously described in the CNS, where EGFR positivity was found predominantly in the motor area compared to the somatosensory area [46], it is tempting to speculate a differential role of EGFR in motor neurons compared to sensory neurons in PNS, although no data are yet present. The expression of EGFR in neurons is high at birth and then decreases during development [134,135]. So, on one hand, the increased EGFR degradation observed in CMT2B patients could lead to an insufficient signaling in the sensory neurons and thus to the loss of the EGFR neurotrophic effect. On the other hand, the excessive signaling due to the inhibition of EGFR degradation observed in the presence of RAB7^K126R^ mutation, could be detrimental for motoneurons. Although the importance of EGFR in promoting neurite outgrowth is known, it was also demonstrated that EGFR activation in astrocytes after spinal cord injury triggers astrocyte activation and this is damaging for neurons as they inhibit axonal regeneration [81]. In the PNS this has not been demonstrated but it could be that different glia cells could play a similar role. The potential negative effect of EGFR in motor neuron regeneration is corroborated by the absence of nerve regeneration at nerve biopsy in our patient with RAB7^K126R^ mutation [130]. This suggests that EGFR expression levels must be finely regulated in the nervous system and this is important to preserve regenerative capability of neurons. Also, the fact that in the predominantly sensory CMT2B there is increased EGFR degradation and thus decreased EGFR amount [131,132,133], while in the motor CMT2B there is inhibited EGFR degradation and consequently increased amount of EGFR [130] suggesting that the phenotypic differences of the disease could be related to EGFR. Further work will be necessary to clarify this issue.

## 5. EGFR Functions after Injury and Its Role in Regeneration

Axonal regeneration in the CNS is inhibited by several inhibitory molecules released in the CNS microenvironment after injury. Among these, chondroitin sulphate proteoglycans (CSPGs) are released by glia cells, myelin associated glycoprotein (MAG) by myelinating glia and oligodendrocyte myelin glycoprotein (OMgp) by oligodendrocytes [136]. Furthermore, astrocytes become reactive producing a glial scar following neuronal cell death induced by injury [137]. Therefore, to make regeneration possible in the CNS, it is necessary to limit the release of these inhibitory molecules and to avoid astrocytes activation.

Several studies indicate that EGFR inhibits regeneration in the CNS through several mechanisms. It was demonstrated that inhibitory molecules such as CSPG and OMgp activate EGFR triggering its rapid phosphorylation and administration of EGFR inhibitors in injured optic nerves of mice stimulates axon regeneration, thus suggesting that EGFR activation contributes to prevent CNS regeneration [138]. According to this negative role of EGFR towards regeneration, Liu and coworkers showed that optic nerve injury induces EGFR activation in astrocytes which become reactive possibly contributing to the glial scar. Intriguingly, EGFR inhibition prevents astrocytes’ activation [84]. Another work demonstrated that infusion of a EGFR inhibitor in the injured spinal cord of rats leads to an improvement of motor, sensory and bladder functions [139]. Moreover, both EGFR inhibitors and genetic deletion are able to promote axonal regeneration in the presence of CSPG and fibrinogen [140]. However, another study demonstrated that the effect of EGFR inhibitors on axonal regeneration is independent from EGFR activation but it is due to off-target effects since these chemicals induce glia and neurons to produce neurotrophins leading to Trk-dependent neurite outgrowth [141]. This apparently controversial result could be due to the use of siRNA, which could attenuate but not eliminate EGFR expression, so residual EGFR could still inhibit neurite outgrowth.

EGFR inhibition has a positive effect also in ameliorating astrogliosis. This process characterizes spinal cord injury in response of which astrocytes become hypertrophic and proliferate creating an unfavorable environment to regeneration [142]. It was demonstrated that EGFR inhibition reduces astrogliosis and the release of proinflammatory cytokines in vitro and also CSPGs production and scar formation in vivo, limiting demyelination and neuronal cell death [143].

Spinal cord injury is also able to activate proliferation of NSCs, which migrate from the central canal to the site of damage [144,145]. The mechanism behind this event has to be clarified. Recently, it was demonstrated that VEGF, released following spinal cord injury because of destruction of the vascular system, is able to induce NSCs proliferation through EGFR-VEGFR2 signaling while spinal injection of EGFR or VEGFR2 inhibitors prevents NSCs activation [146].

Several studies faced the role of EGFR in remyelination. Transgenic mice subjected to unilateral lysolecithin (LPC)-induced demyelination of the corpus callosum and in which EGFR is preferentially overexpressed in the oligodendrocytes lineage show increased number of oligodendrocytes progenitors during the 7 days after the damage compared to wild-type mice [90]. The same work shows that mice overexpressing EGFR display more remyelinated axons and thicker myelin layers 21 days after lesion, compared to wild-type mice remyelination [90]. Therefore, EGFR overexpression in oligodendrocytes lineage after demyelinating lesion of the corpus callosum promotes OPCs’ pool expansion and axon remyelination [90].

Different is the case of spinal cord injury. In a mouse model of spinal cord injury, it was demonstrated that EGFR inhibition in NG2^+^ glial progenitors correlates with their acquisition of neuronal phenotype and with an improvement of motor skills of injured mice [147]. Considering that the ability of glial cells to differentiate into neuronal lineage is already known [148], these data suggest that glial cells could be stimulated to differentiate into neurons inhibiting EGFR and this could be important for neuronal replacement after spinal cord injury. According to these findings, another study shows that the injection of EGFR inhibitor PD168393 in the injured mice spinal cord increases myelination, protects oligodendrocytes and OPCs from apoptosis and reduces activation of astrocytes, microglia and macrophages [149]. Therefore, local EGFR inhibition could be a therapeutic approach after spinal cord injury to promote regeneration.

These apparently contradictory results could be explained considering the different EGFR expression pattern in the CNS. In the normal optic nerve, retinal ganglion cells (RGC) axons do not show EGFR activation while astrocytes, oligodendrocytes and microglia are strongly pEGFR immuno-positive in vivo [150]. On the contrary, it was demonstrated a constitutive activation of EGFR in cultured RGC indicating that this event is not physiological [80,138,150,151]. When CNS injuries occur, EGFR is activated in astrocytes, being involved in the formation of a glia-collagen scar at the lesion site, but not in neurons [80,81]. Indeed, after optic nerve crush, pEGFR levels increased in astrocytes, microglia, macrophages and oligodendrocytes but not in the RGC axons [152]. So, it is unlikely that EGFR inhibitors act directly on neurons to stimulate axonal growth but it is more likely an effect on glia and in particular on astrocytes, limiting the release of inhibitory molecules produced when they become reactive, following EGFR activation [84,153]. Moreover, adult rat astrocytes produce neurotrophic factors (NTF) in vivo and in vitro if they are treated with EGFR inhibitors [150] and it is known that NTF are important to stimulating neurite outgrowth but also to blocking inhibitory signaling [154]. Indeed, the effect of the EGFR inhibitors on regeneration is abrogated by Trk blockade indicating that NTF release by glia is required for axonal regeneration [152].

Furthermore, another study showed EGFR overexpression in cultured rat neurons increase neuron protein β-tubulin and neurofilament and axon protein tau [155], while other studies showed that EGFR inhibition stimulates axonal regeneration. The opposite results could be due to the different targeted cell types since the former study focused on the effect of EGFR on neurons itself while in the others an off-target effect on glia can occur. Xu and coworkers showed that EGFR overexpression in cultured rat neurons activates mammalian target of rapamycin (mTOR) which leads to the production of factors that contribute to axonal regeneration [155]. In adults mTOR is inactivated by phosphatase and tensin homolog (PTEN) and an emerging hypothesis proposes that the CNS does not regenerate in adults because of mTOR inactivation [156].

Altogether these data indicate that EGFR is a negative regulator of regeneration since EGFR inhibition ameliorates astrogliosis, stimulates astrocytes to produce NTF and limits the release of inhibitory molecules, having a secondary effect on neurons stimulating axonal regeneration. On the contrary, EGFR overexpression in oligodendrocyte lineage stimulates remyelination after demyelinating lesion in the corpus callosum suggesting that the effects of EGFR are cell-type dependent. Considering that EGFR expression levels are high during development and then they reduce after birth, EGFR hyperactivation in the adult seems to have negative effects as demonstrated by astrocyte activation following injury, which inhibits neuronal regeneration.

## 6. EGFR in Nervous System Diseases

Since EGFR is heavily involved in signaling and in the regulation of proliferation, it has been found mutated or overexpressed in a number of tumors and it now represents the target for several therapeutic strategies against cancer [157]. As EGFR is also involved in the development and in the maintenance of the nervous system, alteration in its signaling is associated with the onset of several neurological diseases, such as, for instance, Parkinson’s disease, Alzheimer’s disease and amyotrophic lateral sclerosis.

### 6.1. Parkinson’s Disease

Patients suffering from Parkinson’s disease (PD) show motor dysfunctions with progressive neurodegeneration involving dopaminergic neurons of the midbrain [158]. Interestingly, postmortem brains of patients display diminished levels of both EGF and EGFR compared to control subjects [159]. The EGF reduction was confirmed in rat PD models produced through cytotoxic lesion of the substantia nigra dopaminergic neurons, demonstrating that dopaminergic lesions reduce EGF content and limit EGFR activation [159]. Considering the important neurotrophic functions of EGF, the progression of the dopaminergic degeneration in PD patients could be due to reduced EGFR signaling.

**Figure 7:**
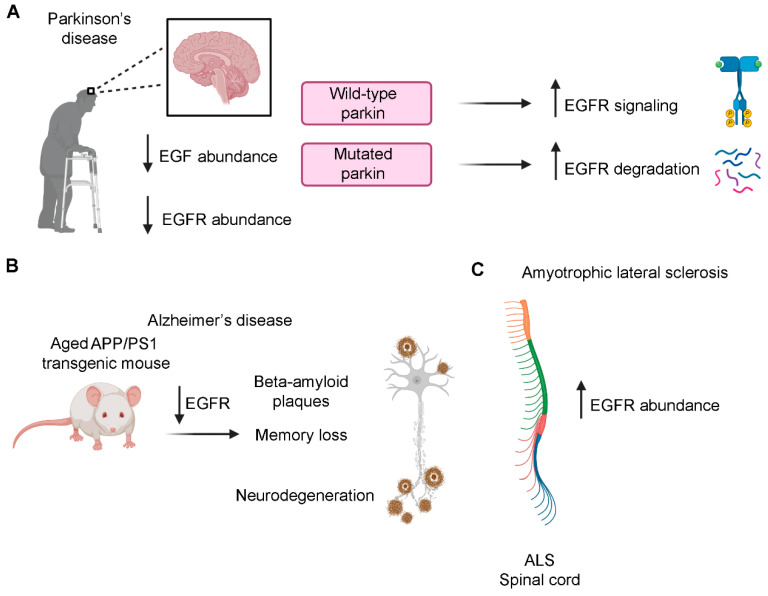
EGFR is associated with neurodegenerative diseases. (**A**) Postmortem brains of patients with Parkinson’s disease show reduced expression of EGF and EGFR. Wild-type parkin sustains EGFR signaling while parkin’s mutations, which are associated with a recessive form of Parkinson’s disease, lead to increased EGFR degradation. (**B**) Aged APP/PS1 double transgenic mice that show plaque formation and memory loss, the hallmarks of Alzheimer’s disease, have reduced EGFR expression that can contribute to neurodegeneration. (**C**) EGFR mRNA is overexpressed in the spinal cord of amyotrophic lateral sclerosis (ALS) patients.

Furthermore parkin, which is mutated in an autosomal recessive form of PD, normally prevents EGFR internalization and degradation, sustaining EGFR signaling [160]. In cultured fibroblasts derived from parkin knockout mice, used as a PD model since pathogenic parkin mutations are related to a loss of the protein functions, EGFR internalization and degradation are higher compared to wild-type fibroblasts [161]. Moreover, this is associated with decreased EGF-induced Akt activation while loss of parkin has no influence on Erk phosphorylation. The reduced Akt signaling observed in parkin null fibroblast has been confirmed in parkin null synaptosomes indicating that parkin specifically regulates Akt signaling in synaptosomes and suggesting that the loss of parkin may lead to dopaminergic neurons degeneration because of the inefficient Akt signaling due to increased EGFR degradation (Figure 7A) [161]. Another gene mutated in an autosomal recessive form of PD is DJ-1 and interestingly, it has been demonstrated that reduced levels of DJ-1 protein are associated with impaired PI3K/Akt signaling in *Drosophila* and this is accompanied by increased ROS production and degeneration of dopaminergic and photoreceptor neurons [162], strengthening the hypothesis that PI3K/Akt signaling impairment could be a common molecular event for PD pathogenesis.

In some familial and sporadic forms of PD the gene encoding for leucine-rich repeat kinase 2 (LRRK2) was found to be mutated [163,164]. Notably, pathogenic LRRK2^G2019S^ variant causes a delay in EGFR degradation but also a defect in EGFR recycling [165]. LRRK2^G2019S^ inactivates RAB8A and this is associated with RAB7A reduced activity and with the appearance of RAB7A-positive tubular structures in cells expressing pathogenic LRRK2 and in fibroblasts derived from patients carrying the LRRK2^G2019S^ mutation [166].

These results suggest that the alterations of EGFR endolysosomal trafficking and consequently of its signaling, following loss of function of proteins important for EGFR intracellular trafficking, could be detrimental for dopaminergic neurons and therefore relevant for the onset and progression of Parkinson’s disease [165]. However, the EGFR diminished levels observed in postmortem brains of PD patients could also be a secondary effect of the loss of dopaminergic neurons due to mutations in genes identified as causative of this pathology or to environmental factors that seem to be related to the onset of PD. Therefore, alterations in EGFR expression and signaling could only contribute to neurodegeneration and not be the leading cause of the onset of PD although further studies are necessary to clarify this issue. In any case, it would be important to establish if increasing EGFR levels could be beneficial in order to revert, even partially, the pathogenic phenotype.

### 6.2. Alzheimer’s Disease

Alzheimer’s disease (AD) is a multiple-factor disease characterized by progressive cognitive impairment and represents the most common cause of dementia [167]. An early-onset familial form of Alzheimer’s disease is due to mutations in genes encoding presenilin proteins [168]. The mechanisms by which mutations in presenilin 1 (PS1) are responsible for the pathogenesis of AD is still unclear. Two hypotheses have been formulated attempting to clarify this issue: the amyloid hypothesis and the presenilin hypothesis. According to the amyloid hypothesis, the production of Aβ42 is increased because of PS1 mutation that leads to enhanced APP processing and excessive production of Aβ42 (gain of function hypothesis) [169]. On the contrary, the presenilin hypothesis is based on the fact that Aβ42 overproduction alone is not sufficient to initiate neurodegeneration in mice [170,171,172] while neurodegeneration is produced by conditional inactivation of presenilins in adult mice brains, increasing the Aβ42/Aβ40 ratio [173,174,175]. Moreover, PS1 is important for memory, learning and neuronal survival so the latter hypothesis based on a loss of PS1 function in AD could better explain AD pathogenesis [176,177]. Interestingly, a correlation exists between PS1 and EGFR, as PS1-null primary cortical neurons from mice show a strong decrease of EGFR abundance and this effect is neuron specific, indicating that neuronal EGFR is transcriptionally regulated by PS1 [178]. The same study shows that ERK and Akt activation followed by EGF treatment is reduced in PS1-null cortical neurons. As EGF exerts neuroprotection through Akt and Erk activation [179,180], to evaluate if PS1 loss affects EGF-dependent neuroprotection, neuronal cultures were treated with glutamate to evaluate excitotoxicity (toxicity due to excessive activation). Indeed, glutamate receptors are important for neuronal functions, but their excessive activation is responsible for neuronal cell death and this occurs also in AD [181]. EGF treatment in wild-type neurons is able to decrease glutamate-induced cell death while in PS1-null neurons administration of EGF is ineffective to increase cell viability, indicating that PS1 is necessary to EGF-induced neuroprotection against excitotoxicity [178]. Importantly, the ability of EGF to activate EGFR, and therefore Akt and Erk, increasing neuroprotection from excitotoxicity following glutamate administration, is restored by expression of exogenous EGFR in PS1-null cortical neurons indicating that PS1 regulates EGFR expression in EGF neuroprotection [178]. PS1 has γ-secretase dependent and independent functions [182,183] and following treatment of primary cortical neurons with a γ-secretase inhibitor, it was demonstrated that γ-secretase does not influence neuronal EGFR [178]. Moreover, a hallmark of this neurodegenerative disease is memory loss induced by amyloid-β (Aβ) oligomers’ accumulation (plaques) [184], which are produced from membrane protein APP cleaved by β and γ-secretases [185]. EGFR is present in the cerebral cortex and hippocampal plaques of patients affected by Alzheimer’s disease [186]. A treatment for Alzheimer’s disease could be based on the possibility to reduce Aβ-oligomers production. Aβ42-expressing *Drosophila melanogaster* is a useful animal model to study Alzheimer‘s disease [187]. In these animals, the treatment with two EGFR inhibitors, erlotinib and gefinitib, ameliorates Aβ42 induced-memory loss but it does not rescue neuronal loss [188]. The fact that EGFR inhibitors could be helpful in the treatment of AD seems to be in contrast with the role of this receptor in neurodegenerative disease since in PD and in other studies regarding AD, EGFR-altered signaling could contribute to neurodegeneration. However, another paper of the same authors shows that EGFR levels are increased in young flies expressing human pan-Aβ42 and showing early stages of AD phenotypes while in aged flies showing late stages of AD phenotype, EGFR levels are strongly reduced and this leads to neuronal degeneration, corroborating the importance of the role of EGFR in sustaining neurons [189].

Moreover, it was demonstrated that APP/PS1 double transgenic mice, which show plaque formation and Aβ-induced memory loss, recover memory following gefinitib treatment [188]. Furthermore in hippocampal tissues of mice, later, the same group demonstrated that EGFR levels decreased with age [189] (Figure 7B). The authors also demonstrated the direct binding of Aβ-oligomers to EGFR since Aβ42 peptides were pulled down with EGFR in COS-7 cells [188]. Even though the demonstration of the interaction between Aβ-oligomers and EGFR is intriguing, further studies are necessary to clarify its involvement in AD. Indeed, it must be considered that, because of the use of non-neuronal systems, data may not reflect what really happens in the nervous system. For example, Bruban and coworkers demonstrated that PS1-null primary cortical neurons of mice have reduced EGFR expression while distinct immortalized mouse embryonic fibroblast cell lines show a great variability in EGFR levels regardless of the PS1 genotype [178]. Furthermore, while memory defects characterize early stages of AD, neurodegeneration appears in the late stages. Consistently, while in the early stages of the disease oligomers activate EGFR inducing memory loss, in the late stages of AD EGFR levels are reduced leading to neurodegeneration [189]. This bidirectional regulation of EGFR by Aβ42 oligomers could be considered for therapeutic strategies against AD.

Another EGFR inhibitor, afatinib, acts inhibiting EGFR tyrosine kinase activity [190]. Recently, it was demonstrated that this drug is able to reduce neuroinflammation and prevents activation of cultured astrocytes [191]. Astrocytes overexpress EGFR in Alzheimer’s disease [192]. Therefore, blocking EGFR activation in astrocytes could be beneficial for the treatment of neuroinflammation, which characterizes CNS neurodegenerative diseases including Alzheimer’s disease although this has still to be proved.

### 6.3. Amyotrophic Lateral Sclerosis (ALS)

ALS is a neurodegenerative disorder affecting motoneurons associated with a rapid progressive paralysis, and patients’ death comes 2–5 years after diagnosis in the 80% of the cases [193]. It has been demonstrated that EGFR mRNA is overexpressed in the spinal cord of ALS patients (Figure 7C) [194]. For this reason, it was hypothesized that EGFR inhibition could be helpful in the treatment of this pathology. A mouse model of ALS with the SOD1^G93A^ mutation, treated with EGFR inhibitor erlotinib, showed a delay in symptom progression but their lifespan was not extended and this drug does not seem to be effective in protecting motor synapses [195]. Erlotinib’s effect not on motoneurons but on other cell types such as glia could explain this result, but other studies are necessary to clarify this issue. Moreover, it must be considered that EGFR mRNA is overexpressed in ALS patients but data on EGFR protein abundance are not yet available and thus the inefficacy of the treatment with EGFR inhibitors could be due to protein levels not reflecting mRNA levels. A possibility could be represented by increased EGFR protein degradation which could correlate with the low EGFR signaling already seen in AD and PD and that could lead to neurodegeneration.

## 7. Conclusions

In this review, we discussed the main roles of EGFR in the nervous system. Notably, EGFR is important for neural stem cells’ pool maintenance, for astrocytes’ maturation and functions, for oligodendrogenesis and neurite outgrowth in the CNS. In the PNS, the functions of EGFR are less-understood at the molecular level although its importance in PNS development is clear.

Several reports indicate that EGFR inhibition ameliorates astrogliosis following injuries while it has a protective role towards oligodendrocytes, stimulating remyelination. In fact, EGFR inhibition in astrocytes limits the release of inhibitory molecules and stimulates the production of neurotrophic factors, having, as a secondary effect on neurons, the simulation of axonal growth. However, axonal growth is also stimulated in neurons directly by EGFR overexpression. Therefore, the apparently contradictory results on the role of EGFR in axonal regeneration depend mainly on the targeted cell type. In any case, EGFR seems to have a strong impact on axonal regeneration although its role should be also evaluated together with other neurotrophic factors that act on the nervous system to have a more clear scenario of the role of EGFR compared to other growth factors.

Importantly, alterations of EGFR trafficking and signaling are associated with the onset and progression of several neurodegenerative diseases suggesting that modulation of EGFR expression or signaling could be useful to stimulate regeneration or counteract neurodegeneration.
